# Prevalence of Hypertension, Diabetes, and Other Cardiovascular Disease Risk Factors in Two Indigenous Municipalities in Rural Guatemala: A Population-Representative Survey

**DOI:** 10.5334/gh.1171

**Published:** 2022-11-22

**Authors:** Eric Steinbrook, David Flood, Joaquin Barnoya, Carlos Mendoza Montano, Ann C. Miller, Peter Rohloff

**Affiliations:** 1 University of Michigan Medical School, University of Michigan, Ann Arbor, MI, USA; 2 Centro de Investigación en la Salud Indígena, Wuqu’ Kawoq, Tecpán, Guatemala, US; 3 Instituto de Nutrición de Centroamérica y Panamá, INCAP, Guatemala City, Guatemala Unidad de Cirugia Cardiovascular, Guatemala City, Guatemala, US; 4 Unidad de Cirugia Cardiovascular de Guatemala, Guatemala City, Guatemala, US; 5 Integra Cancer Institute, Guatemala City, Guatemala, US; 6 Department of Global Health and Social Medicine, Harvard Medical School, Boston, Massachusetts, USA; 7 Division of Global Health Equity, Brigham and Women’s Hospital, Boston, Massachusetts, USA

**Keywords:** Cardiovascular risk factors, diabetes, Guatemala

## Abstract

**Background:**

Nearly 50% of Guatemalans are Indigenous Maya, yet few studies have examined the prevalence of modifiable cardiovascular disease (CVD) risk factors in Indigenous Maya populations. Therefore, we sought to estimate the prevalence of modifiable CVD risk factors in two Indigenous Maya areas in Guatemala.

**Methods:**

We conducted, between June 2018 and October 2019, a population-representative survey of adults aged 18 years and older in two rural Indigenous Maya municipalities in Guatemala. Our primary outcomes were five modifiable CVD risk factors: diabetes, hypertension, obesity, smoking, and alcohol use. We estimated the crude and age-standardized prevalence of each outcome. We also constructed multivariable logistic regression models to assess prevalence over covariates including age, sex, education level, ethnicity, and poverty. Sampling weights adjusted for nonresponse, and appropriate survey commands were used in all analyses.

**Results:**

The crude prevalence of diabetes was 12.5% (95% confidence Interval [CI] 9.6% to 16.1%), hypertension 20.3% (95% CI 17.1% to 23.9%), obesity 23.7% (95% CI 19.4% to 28.6%), smoking 10.7% (95% CI 7.8% to 14.5%), and high alcohol use 0.9% (95% CI 0.5% to 1.6%). Age-standardized prevalence of each outcome was similar to the crude prevalence. The prevalence of multiple CVD risk factors increased between the age groups 18–29 years and 50–59 years before decreasing among older age groups. Men had twenty-fold higher smoking prevalence than women (20.5% vs. 1.2%, respectively) and women had nearly double the age-adjusted prevalence of obesity as men (30.1% vs. 17.0%, respectively).

**Conclusion:**

There is a substantial prevalence of modifiable CVD risk factors in rural, Indigenous populations in Guatemala, in particular hypertension, diabetes, obesity (among women), and smoking (among men). These findings can help catalyze policy and clinical investments to improve the prevention, management, and control of CVD risk factors in these historically marginalized communities.

## Introduction

Cardiovascular disease (CVD) is the leading cause of global mortality [1]. Studies in multiple countries indicate a greater burden of CVD and higher prevalence of CVD risk factors in Indigenous compared to non-Indigenous populations [2,3,4,5,6,7]. As the incidence of CVD risk factors in Indigenous populations rises [8], CVD disparities may become further entrenched. Indigenous populations worldwide have important differences, but they share common structural factors that give rise to CVD including losses of land and environmental degradation attributable to colonialism, underinvestment in health care systems, and profound socioeconomic marginalization, among other factors [9,10].

Guatemala is a Central American country of 17 million people of whom nearly 50% are Indigenous Maya [11]. CVD is now the leading cause of death in Guatemala [12], and there has been limited progress towards decreasing the burden of CVD in the general population [13]. Many studies in Guatemala show that, compared to non-Indigenous groups, Indigenous people experience inequities in maternal and child health outcomes and health care access [14,15,16,17,18]. However, few studies have examined the epidemiology of CVD risk factors in Indigenous communities in Guatemala [19]. A 2015 subnational survey based on the World Health Organization’s STEPwise approach to NCD risk factor surveillance (STEPS) did not sample Indigenous areas outside the capital [19]. Similarly, the Central America Diabetes Initiative (CAMDI) survey conducted between 2003 and 2006 focused on an urban, non-Indigenous municipality in the capital region [20]. To our knowledge, the only attempt to estimate prevalence of CVD risk factors among both men and women at the national level was a 2010 study conducted by the country’s public medical school, but this study did not report results by ethnicity [21]. In predominantly Indigenous areas of Guatemala, only a few small studies have been conducted [22,23,24].

Knowledge of the prevalence of CVD risk factors in Indigenous populations in Guatemala can identify important disparities and inform future policy and program initiatives. Therefore, the objective of this analysis was to estimate the prevalence of CVD risk factors using data from a population-representative survey in two predominantly Indigenous municipalities in Guatemala.

## Methods

### Study design

We conducted a cross-sectional, population-representative survey between June 2018 and October 2019 in two rural municipalities in Guatemala. We previously have published the survey’s primary outcomes relating to chronic kidney disease (CKD) [25], and in this analysis we report secondary outcomes of modifiable CVD risk factors. In designing the survey, we chose the municipalities of Tecpán, Chimaltenango (estimated population: 85,000) and San Antonio Suchitepéquez, Suchitepéquez (estimated population: 52,000) because they both have predominantly Maya Indigenous populations but have different potential environmental exposures for CKD. The institutional review boards of Maya Health Alliance (WK 2018 001), the Institute for Nutrition of Central America and Panama (CIE REV 075/2018), and Partners Healthcare (2017P002476) approved the study.

### Study sample

The survey sampled non-pregnant adults aged 18 years or older living in each municipality. We planned to sample at least 700 people from 350 households. As noted above, our primary goal in designing the survey was to measure CKD in Indigenous communities in Guatemala. We calculated sample size by hypothesizing a 10% prevalence of CKD, a margin of error of 0.35, 10% refusals, and a design effect of 2 to account for household clustering with at least two eligible residents per household [25].

We have previously described our novel satellite-based sampling methodology [26]. Briefly, given the lack of a unique home address system or up-to-date national census at the time of the survey, we were unable to use traditional sampling methods using population-based enumeration areas. Instead, we implemented the Geo-Sampler program developed by EpiCentre to randomly select a sample of 350 structures across the two sites using satellite images. This method avoided the time-intensive process of cataloging structures and improved statistical efficiency by using a simple random sample instead of two- or three-stage cluster sampling. The data collection team approached each selected structure and invited all eligible residents to participate in the survey.

### Data collection

The data collection team interviewed participants using a survey containing items on sociodemographic information, medical history, and physical and biochemical measurements. Interviews were conducted in either Spanish or a Mayan language depending on respondent preference. For physical measurements, height, weight, and blood pressure were taken following standardized methods [25,27]. We estimated height and weight as the mean of the three measurements using a Seca portable stadiometer (Models 123 and 2013) and Tanita portable digital scales (Model BC-558), respectively. We measured blood pressure using a Omron Series 7 digital automated cuff, which has been clinically validated through an independent review process [28]. We measured blood pressure after 15 minutes rest; the mean of the second and third measurements were used in this analysis [29] (Appendix Supplementary Methods). For biochemical measurements, we collected venous blood in the field, froze samples at –20°C, and then transported samples to a reference laboratory at the Institute for Nutrition of Central America and Panama. Glycated hemoglobin (HbA1C) levels were measured using the Roche Cobas C111 analyzer. Additional details on data collection and processing can be found in our prior report [25].

### Outcomes

Our primary outcomes for this analysis were five modifiable risk factors for CVD: diabetes, hypertension, obesity, smoking, and alcohol use. These outcomes were chosen based on the overlap between data available in our survey and the modifiable CVD risk factors defined in the Prospective Urban Rural Epidemiology (PURE) study, a longitudinal cohort study of CVD risk factors and mortality among adults in 21 low-, middle-, and high-income countries. The PURE definitions of CVD risk factors align with most clinical guidelines [30]. We defined our outcomes in line with the PURE study with a few small differences as noted below.

#### Diabetes

We defined diabetes as either glycated hemoglobin (HbA1c) ≥6.5% or a self-reported history of diabetes [31,32]. Our definition of diabetes differed from the PURE study, which used fasting glucose rather than HbA1c as the diabetes biomarker.

#### Hypertension

Hypertension was defined as either systolic blood pressure (SBP) ≥140 mmHg, diastolic blood pressure (DBP) ≥90mmHg, or a self-reported history of hypertension or self-reported use of anti-hypertension medications. Guidelines from the World Health Organization (WHO) package of essential non-communicable (PEN) disease interventions for primary health care recommend these blood pressure thresholds [33]. In a sensitivity analysis, we also used an alternative threshold of SBP ≥130 mmHg or DBP ≥80 mmHg as recommended in the 2017 ACC/AHA guidelines [34] and 2018 primary care guidelines from the Guatemalan Ministry of Health [35]. Our definition differed from the PURE study, which uses SBP ≥140 mmHg or DBP ≥ 90 mmHg or taking medication for Hypertension and does not consider self-reported history of hypertension.

#### Obesity

We defined categories of body mass index (BMI) according to conventions used by the WHO and US National Institutes of Health [36,37]. We defined underweight as BMI <18.5 kg/m^2^, healthy weight as BMI ≥18.5 kg/m^2^ but <25 kg/m^2^, overweight as ≥25 kg/m^2^ but <30 kg/m^2^, and obesity as ≥30 kg/m^2^. We used obesity as a proxy for abdominal obesity, which differs from the PURE study’s use of waist-to-hip ratio.

#### Smoking

Smoking was self-reported using a tobacco use frequency questionnaire with categories of current daily, current less than daily, past, and never smoker. We defined smoking status as current tobacco use and, in a sensitivity analysis, as any lifetime tobacco use.

#### Alcohol use

Alcohol use was also self-reported using a frequency questionnaire. Questions included categorical variables for days of consumption per week over the last 12 months. We defined high alcohol consumption as alcohol use more than two days per week. While the PURE study quantified alcohol use in terms of number of drinks per week, we did not quantify the number of drinks consumed and therefore defined alcohol use in terms of frequency alone.

### Statistical Analysis

First, we generated descriptive statistics in the overall sample and the gender-stratified sample. Second, we estimated the crude and age-standardized prevalence of each outcome. Age-standardized prevalence of outcomes was estimated using direct standardization to the WHO reference population [38]. Third, we estimated the prevalence of co-occurring outcomes of modifiable CVD risk factors by age. Fourth, we constructed multivariable logistic regression models to estimate the age- and sex-adjusted prevalence of each outcome. Model covariates included age, sex, education level (secondary or higher, primary, or no education), ethnicity (Indigenous or non-Indigenous), and poverty. Poverty was defined as the probability a household was below the national poverty line [39]. Age was specified as a continuous variable with a linear and squared term. We computed prevalence as predicted probabilities from the models and computed the absolute difference in prevalence as the average marginal effect relative to the reference category indicated [40]. We used sampling weights adjusted for differential nonresponse among men and women in all calculations and Stata’s *svy* commands to account for stratification between the two municipalities. We used Stata version 17.0 for all analyses.

## Results

### Participant characteristics

Out of 806 participants surveyed, most were women (526, 65.3%). Table 1 presents characteristics of the study population. Overall, 16.2% (95% confidence interval [CI] 11.5% to 20.8%) were living below the national poverty line, 33.4% (95% CI 27.7% to 39.7%) had no formal education, and 79.4% (95% CI 72.0% to 85.3%) of the participants identified as Indigenous. 37.2% (95% CI 31.6% to 43.0%) were between the ages of 18–29 years. Sociodemographic characteristics did not differ significantly between men and women.

**Table 1:** **Participant characteristics.** Prevalence estimates were survey weighted with 95% confidence intervals in parentheses. Percentages reflect the percent of participants within a given column (i.e., column totals). CI = confidence interval.


	TOTAL (N=806)	MALE (N=280)	FEMALE (N=526)

CHARACTERISTIC	PREVALENCE, % (95% CI)		

Age (years)			

18–29	37.2 (31.6 to 43.0)	34.7 (27.7 to 42.5)	39.6 (33.2 to 46.3)

30–39	16.2 (12.5 to 20.6)	16.2 (11.2 to 23.0)	16.1 (12.1 to 21.2)

40–49	18.3 (13.7 to 24.1)	19.0 (12.9 to 27.2)	17.6 (13.7 to 22.3)

50–59	14.2 (10.9 to 18.2)	13.5 (9.5 to 19.0)	14.7 (10.2 to 20.9)

60–69	7.7 (5.2 to 11.3)	8.2 (4.4 to 14.9)	7.2 (4.6 to 11.0)

≥70	6.5 (4.3 to 9.7)	8.3 (4.8 to 14.0)	4.7 (2.9 to 7.6)

Education level			

Secondary or higher	28.9 (22.6 to 36.1)	32.5 (24.6 to 41.5)	25.5 (19.7 to 32.2)

Primary	37.7 (32.9 to 42.7)	40.6 (33.7 to 47.9)	34.9 (30.0 to 40.0)

No education	33.4 (27.7 to 39.7)	27.0 (20.1 to 35.2)	39.7 (33.6 to 46.1)

Ethnicity			

Indigenous/Maya	79.4 (72.0 to 85.3)	79.7 (70.7 to 86.5)	79.1 (71.8 to 84.9)

Non-Indigenous	20.6 (14.7 to 28.0)	20.3 (13.5 to 29.3)	20.9 (15.1 to 28.2)

Below national poverty line	16.2 (11.5 to 20.8)	17.0 (10.7 to 23.2)	15.4 (11.3 to 19.4)


### Prevalence of modifiable CVD risk factors

**Figure 1:**
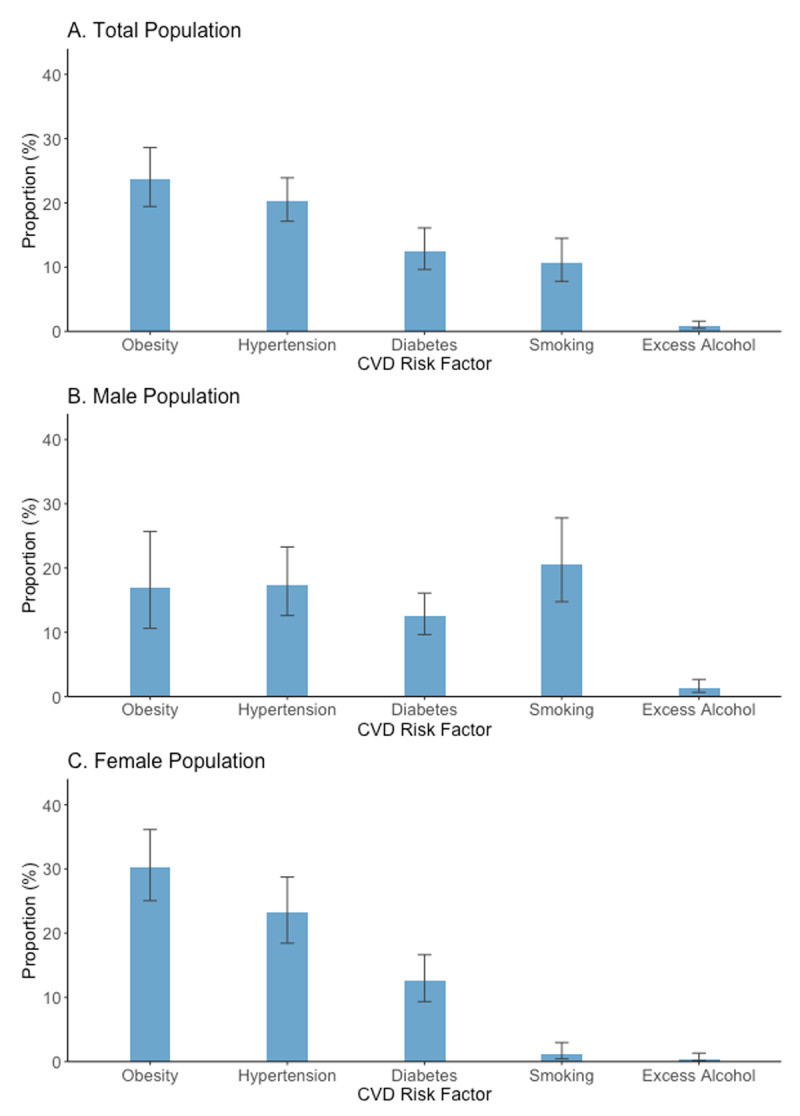
**Crude prevalence of modifiable cardiovascular disease risk factors**. Prevalence estimates were survey weighted. Obesity was defined as body mass index of ≥30 kg/m^2^. Hypertension was defined as ≥140/90 mmHg or self-report of diagnosis or self-reported use of anti-hypertension medication. Diabetes was defined as HbA1c ≥6.5% or self-report of diagnosis. Smoking was defined as a response of ‘current’ on a smoking questionnaire with options of never, former, or current. The term ‘crude’ refers to prevalence values in this table that are not age standardized to the WHO standard population. Values underlying this figure are presented in Supplementary Table S1. CVD = cardiovascular disease.

Figure 1 and Supplementary Table S1 show the crude (i.e., non-age standardized) prevalence of modifiable CVD risk factors in the total sample and in subgroups of men and women. The overall prevalence of diabetes was 12.5% (95% CI 9.6% to 16.1%), hypertension was 20.3% (95% CI 17.1% to 23.9%), obesity was 23.7% (95% CI 19.4% to 28.6%), smoking was 10.7% (95% CI 7.8% to 14.5%), and high alcohol use was 0.9% (95% CI 0.5% to 1.6%). The corresponding age-standardized prevalence were diabetes, 12.7% (95% CI 9.9% to 16.1%); hypertension, 21.3% (95% CI 18.2% to 24.8%); obesity, 23.9% (95% CI 19.8% to 28.6%); current smoking, 10.6% (95% CI 7.7% to 14.3%); and high alcohol use, 0.8% (95% CI 0.5% to 1.5%).

### Prevalence of co-occurring modifiable CVD risk factors by age

**Figure 2:**
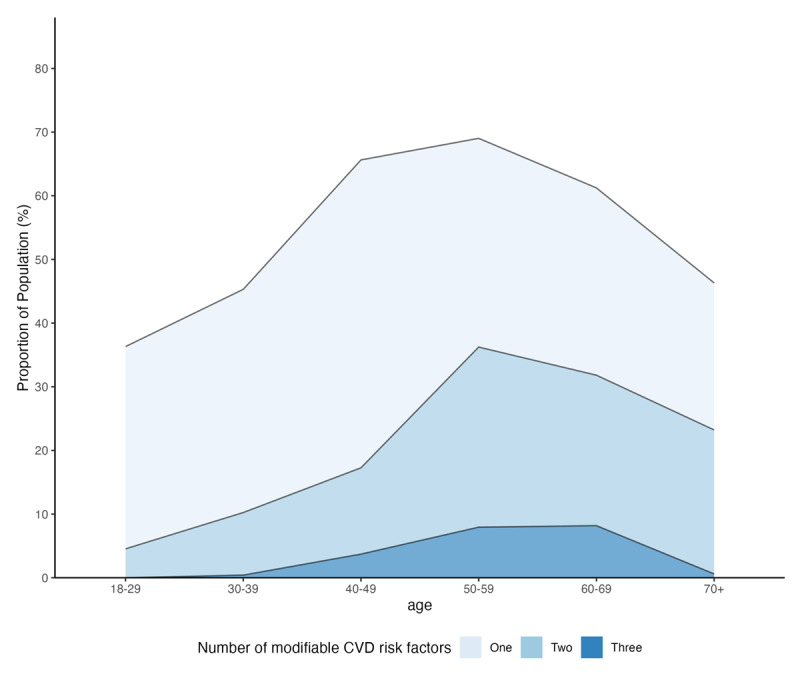
**Crude prevalence of co-occurring modifiable cardiovascular disease risk factors by age**. Prevalence estimates were survey weighted. Obesity was defined as body mass index of ≥30 kg/m^2^. Hypertension was defined as ≥140/90 mmHg or self-report of diagnosis or self-reported use of anti-hypertension medication. Diabetes was defined as HbA1c ≥6.5% or self-report of diagnosis. Smoking was defined as a response of “current” on a smoking questionnaire with options of never, former, or current. The term “crude” refers to prevalence values in this table that are not age standardized to the WHO standard population. Values underlying this figure are presented in Supplementary Table S1. CVD = cardiovascular disease. Given the small prevalence of four comorbidities, we exclude them from the figure.

Figure 2 and Supplementary Table S2 show the crude prevalence of co-occurring modifiable CVD risk factors by age. The prevalence of multiple modifiable CVD risk factors increases between the age groups 18–29 years and 50–59 years before decreasing among older age groups. Among those 50–59 years of age, which was the age group with the highest prevalence of modifiable CVD risk factors, 69.0% (95% CI 55.7% to 79.8%) had one risk factor, 36.2% (95% CI 26.3% to 47.5%) had two, 7.9% (95% CI 3.6% to 16.7%) had three, and 0.4% (95% CI 0.1% to 3.1%) had four risk factors.

### Prevalence of obesity, diabetes, and hypertension across participant characteristics

Table 2 depicts the adjusted prevalence of obesity, diabetes, and hypertension across participant sociodemographic characteristics. Adjusting for sex, the prevalence of each of these risk factors tended to increase in middle age and then decline in older ages. Adjusting for age, the prevalence of obesity was higher in women compared with men. The age- and sex-adjusted prevalence of diabetes was higher among respondents with greater educational attainment. The age- and sex-adjusted prevalence of obesity and hypertension—though not diabetes—was higher among non-Indigenous ethnicity. Respondents who had a higher probability of living below the poverty line were less likely to be obese in the age- and sex-adjusted models, but we observed no differences for the prevalence of diabetes and hypertension by poverty status.

**Table 2:** **Age- and sex-adjusted prevalence of obesity, diabetes, and hypertension across participant characteristics.** ^a^ Adjusted for age (linear and squared term) only. ^b^ Adjusted for sex only. ^c^ Adjusted for age (linear and squared term) and sex. Prevalence was computed as predicted probabilities from the multivariable logistic regression models. Absolute difference in prevalence was computed as the average marginal effect relative to the reference category indicated. Obesity was defined as body mass index of ≥30 kg/m^2^. Hypertension was defined as ≥140/90 mmHg or self-report of diagnosis or self-reported use of anti-hypertension medication. Diabetes was defined as HbA1c ≥6.5% or self-report of diagnosis. All estimates are survey weighted and account for survey design. CI = confidence interval. Ref = reference category.


	OBESITY		DIABETES		HYPERTENSION	

CHARACTERISTIC	PREVALENCE, % (95% CI)	ABSOLUTE DIFFERENCE, % (95% CI)	PREVALENCE, % (95% CI)	ABSOLUTE DIFFERENCE, % (95% CI)	PREVALENCE, % (95% CI)	ABSOLUTE DIFFERENCE, % (95% CI)

Age (years)^a^						

25	16.5 (10.5 to 22.5)	Ref	1.1 (0.1 to 2.2)	Ref	6.2 (2.6 to 9.7)	Ref

35	29.8 (22.6 to 37.0)	+13.3 (7.7 to 18.9)	6.1 (3.1 to 9.1)	+5.0 (2.8 to 7.1)	15.9 (11.5 to 20.3)	+9.7 (6.9 to 12.5)

45	36.8 (28.5 to 45.1)	+20.3 (11.4 to 29.3)	18.0 (12.1 to 23.9)	+16.9 (11.2 to 22.6)	28.7 (22.7 to 34.6)	+22.5 (15.8 to 29.2)

55	34.0 (26.2 to 41.7)	+17.5 (7.8 to 27.2)	30.5 (22.0 to 38.9)	+29.4 (20.6 to 38.1)	38.7 (31.8 to 45.6)	+32.5 (24.0 to 41.0)

65	22.5 (13.2 to 31.9)	+6.1 (–5.1 to 17.2)	34.2 (24.7 to 43.7)	+33.1 (23.2 to 42.9)	42.4 (34.6 to 50.1)	+36.2 (27.2 to 45.2)

75	9.5 (0.4 to 18.5)	–7.0 (–16.5 to 3.6)	26.8 (13.8 to 39.8)	+25.6 (12.6 to 38.6)	38.9 (24.9 to 52.9)	+32.7 (18.9 to 46.6)

Sex^b^						

Male	17.0 (9.4 to 24.7)	Ref	12.2 (7.4 to 16.9)	Ref	16.9 (11.4 to 22.4)	Ref

Female	30.1 (24.8 to 35.3)	+13.0 (4.0 to 22.0)	12.9 (9.4 to 16.4)	+0.8 (–4.8 to 6.3)	23.8 (19.5 to 28.1)	+6.9 (0 to 14.3)

Education level^c^						

Secondary or higher	28.2 (16.6 to 39.9)	Ref	22.5 (14.6 to 30.4)	Ref	17.4 (10.2 to 24.6)	Ref

Primary	24.1 (17.9 to 30.3)	–4.1 (–17.1 to 8.8)	13.6 (7.9 to 19.4)	–8.9 (–18.5 to 0.7)	23.0 (17.4 to 28.7)	+5.6 (–3.3 to 14.5)

No education	20.3 (13.6 to 27.0)	–7.9 (–21.5 to 5.6)	8.3 (5.3 to 11.2)	–14.2 (–22.5 to –5.7)	19.7 (13.9 to 25.4)	+2.2 (–7.9 to 12.4)

Ethnicity^c^						

Indigenous	20.0 (15.7 to 24.3)	Ref	11.4 (8.0 to 14.8)	Ref	16.8 (13.6 to 20.1)	Ref

Non-Indigenous	38.9 (25.9 to 51.8)	+18.8 (5.4 to 32.3)	17.3 (10.8 to 23.9)	+6.0 (–1.2 to 13.1)	34.5 (25.1 to 43.8)	+17.6 (7.5 to 27.8)

Probability under the national poverty line^c^						

0%	22.5 (16.9 to 28.1)	Ref	12.2 (7.9 to 16.5)	Ref	19.6 (15.5 to 23.7)	Ref

50%	29.9 (17.3 to 42.6)	+7.4 (–7.5 to 22.3)	13.2 (7.0 to 19.4)	+1.0 (–7.1 to 9.2)	22.7 (14.2 to 31.3)	+3.2 (–7.1 to 13.4)

100%	7.5 (0 to 16.8)	–15.0 (–22.5 to –4.7)	13.9 (0 to 28.7)	+1.7 (–12.2 to 16.5)	17.4 (8.0 to 26.8)	–2.2 (–11.9 to 7.6)


### Sensitivity analyses

In the sensitivity analysis of hypertension using an alternative threshold of SBP ≥130 mmHg, DBP ≥80 mmHg, or self-reported history of diagnosis or use of anti-hypertensive medication, we observed a prevalence of 37.4% (95% CI 32.6% to 42.4% Supplementary Table S1), which was higher than crude prevalence in our primary analysis of 20.3% (95% CI 17.1% to 23.9%). The overall crude prevalence of current smoking in our primary analysis was 10.7% (95% CI 7.8% to 14.5%); in the sensitivity analysis broadening the definition of smoking to any lifetime tobacco use, we observed a higher prevalence of 32.2% (95% CI 27.8% to 37.0%) and notable gender-based differences (men: 57.8% [95% CI 49.0% to 66.1%]; women: 7.5% [95% CI 5.2% to 10.7%]).

## Discussion

In this population-representative survey of modifiable CVD risk factors among adults in two rural Indigenous municipalities in Guatemala, we found that approximately one in eight individuals had diabetes, one in five had hypertension, one in four were obese, and one in ten were current smokers. We also found that, across all age groups, 40% or more of adults had at least one modifiable CVD risk factor. Our findings reveal the imperative to implement clinical and public health programs to address the substantial burden of modifiable CVD risk factors in Indigenous communities in Guatemala where there is a historic underinvestment in health care infrastructure and programs.

The findings in our subgroup analyses also merit further consideration. First, similar to the 2014–2015 Demographic and Health Survey (DHS) [41], we found that men had nearly twenty-fold higher prevalence of current daily smoking than women (20.5% vs. 1.2%, respectively). Tobacco use may be perceived as a primarily non-Indigenous issue in Guatemala, but our findings suggest that Indigenous populations should be included in smoking reduction initiatives [42]. Second, it was notable that women had nearly double the age-adjusted prevalence of obesity as men (30.1% vs. 17.0%; Supplementary Table S1 shows all BMI categories). The DHS and other national reproductive health surveys collect anthropometric measures only among women [41,43,44], and our study is one of few to report the BMI status of Indigenous men in Guatemala [23]. We speculate that gender differences in obesity in these communities are driven by greater physical activity among men practicing intensive agricultural occupations [45]. Third, we observed a decline in the prevalence of modifiable CVD risk factors among people aged 60 years or older. This finding should be interpreted with caution given the relatively small sample (<15% of the total sample) and the risk of survivorship bias. One potential explanation, however, is that older individuals in these communities were not exposed to as many risk factors for obesity during early and middle adulthood compared to younger individuals. Growing up in a less obesogenic environment may have helped older individuals preserve cardiovascular health during later life, whereas a greater penetration of processed food and more sedentary lifestyles in younger individuals may have increased CVD risk factors [45].

Our study contributes particular value in further understanding the epidemiology of diabetes and hypertension among Indigenous populations in Guatemala. The most well-known national survey program in Guatemala, the DHS series of surveys, does not assess either of these conditions, and there have only been a few prior diabetes or hypertension population surveys in Indigenous areas of the country. Our estimate of a diabetes prevalence of 12.5% was similar to a 2015 survey conducted in Indigenous towns surrounding Lake Atitlán (diabetes prevalence of 13.8%) [24]. However, this 2015 study implemented significantly different methodology than ours, as they used a gridded sampling frame, classified diabetes using HbA1c cut-offs alone (i.e., no consideration of self-reported diabetes diagnosis), and measured HbA1c using a point-of-care device that may have underestimated prevalence [46,47]. Another study conducted in 2012–2013 in the Indigenous municipality of Santiago Atitlán reported a diabetes prevalence of only 3.0%. This study defined diabetes as fasting blood glucose ≥126 mg/dL or use of a glucose-lowering medication [23]. It is not clear if differences based on use of HbA1c or fasting glucose can adequately explain the large variation observed between studies [48]. Other possible reasons for the variation could be intrinsic differences in the Indigenous municipalities sampled or a rapidly rising diabetes prevalence in Indigenous communities in Guatemala in the six years between this survey and ours.

Regarding hypertension in predominantly Indigenous areas of Guatemala, our prevalence of 20.3% was similar to Santiago Atitlán (hypertension prevalence of 18.3%) [23], higher than the Department of Sololá (hypertension prevalence of 12.5%) [22], and higher than a nationally representative survey of women aged 15–49 (hypertension prevalence among Indigenous subpopulation of 12.8%) [43,44]. Overall, the hypertension prevalence in our study was lower than in the STEPS survey in Guatemala City [49] and also lower than modeled national prevalence in Guatemala from the NCD Risk Factor Collaboration [19]. Our sample reflects Guatemala’s younger population age distribution (53% of respondents under 40 years of age), and we found a hypertension prevalence of approximately 40% among respondents aged 55 years and older. Importantly, comparisons of hypertension prevalence across studies are complicated by subtle differences in measurement methods and definitions. For example, we classified all respondents as having hypertension if they self-reported a prior diagnosis—even if they were not taking a blood pressure lowering medication and had blood pressure <140/90 mmHg. Our definition likely led to a slightly higher hypertension prevalence than would have been obtained using an alternative definition that did not include self-reported diagnosis [19,50,51].

Our study was focused on the most common CVD risk factors. However, it is also notable how these shared risk factors can contribute to morbidity and mortality for non-CVD outcomes. For example, in our prior study assessing CKD prevalence, we found that about 4% of respondents had chronic kidney disease (defined as eGFR below 60 ml/min per 1.73 m^2^). Moreover, we observed that most CKD was associated with diabetes and hypertension [25]. CVD risk factors such as diabetes and hypertension contribute to the rapid increase in the number of people with conditions such as cataracts, diabetic retinopathy, and end stage kidney disease in Guatemala [52,53,54].

The policy implication of this study is that both design and implementation of evidence-based policies are needed to address diabetes, hypertension, and other modifiable CVD risk factors among adults in predominantly Indigenous areas in Guatemala. Such policies are important not only because of the sizeable prevalence of these risk factors but also because there is an ethical obligation to pursue health equity for Indigenous populations who have experienced longstanding health disparities [18]. One overarching target is Sustainable Development Goal 3.4, which aims to reduce the rate of premature mortality from non-communicable diseases by one-third by the year 2030 [55]. In Guatemala, there has unfortunately been limited progress toward achieving the target [13,56], and scarce data are available for Indigenous subpopulations. Nevertheless, there is a clear path forward to accelerate health gains by prioritizing two main responses: (a) intersectoral policies like trans-fat bans, sodium reduction, and tobacco control [42]; and (b) targeted investments in the health care system to improve the prevention, management, and control of modifiable CVD risk factors [57,58]. Currently, Guatemala has a non-mandatory declaration encouraging industry to reduce trans fats and salt in processed foods, and a bill on front-labelling processed foods is on standby in Congress. These types of interventions may help to address rapid shifts in the diets in Indigenous areas of Guatemala where processed foods increasingly replace traditional diets comprised of corn, beans, and fresh vegetables and greens [59]. The Guatemalan Ministry of Health has recently signed on to the HEARTS in the Americas program, led by the Pan American Health Organization, with the goal of implementing a population-based, integrated national primary care program to address hypertension, diabetes, and other CVD risk factors. It will be important to ensure that this initiative also reaches the health system in rural and Indigenous communities [60].

Some weaknesses and limitations must be taken into account. First, this was an analysis of a population-representative survey that was powered to assess CKD prevalence and risk factors. Thus, our study did not have available data to assess some important modifiable CVD risk factors including cholesterol, diet, and physical activity. In addition, we had limited precision especially in our subpopulation estimates. Second, the two municipalities we surveyed may not be representative of all rural and Indigenous areas in Guatemala. At the same time, our focus on a smaller number of study municipalities was a strength, as it facilitated rigorous survey methodology using satellite-based sampling of physical structures [26]. In the future, implementation of a national non-communicable disease survey such as those based on the WHO STEPS framework in Guatemala would be a critical step to achieve nationally representative estimates of the CVD risk factor burden.

Third, self-reported smoking prevalence has been shown to significantly underestimate smoking prevalence in rural Guatemala, and our data likely underestimated the true smoking prevalence [61]. Fourth, we used a non-standard definition of excess alcohol use (drinking more than two days per week) as we did not have measures of both frequency and quantity of alcohol use. Fifth, we used HbA1c as a diabetes biomarker rather than fasting glucose. While our use of HbA1c makes it challenging to compare our results with other population surveys both in Guatemala and internationally that use glucose biomarkers, there are also advantages to using HbA1c: it (a) has excellent diagnostic test characteristics for diabetes as typically defined in clinical practice [62], (b) minimizes measurement error that may occur when fasting status is not accurately reported alongside glucose measurements, and (c) is more closely associated with incident CVD than fasting glucose [63]. Finally, we had difficulty recruiting men in our household survey, as illustrated by our sample that consisted of approximately twice as many women as men. While we accounted for this differential non-response in our survey analysis, there remains the possibility that male respondents differed from the overall population of men in study municipalities.

## Conclusion

In conclusion, this population-representative survey in two rural Maya Indigenous municipalities of Guatemala offers the most up-to-date, methodologically rigorous data on modifiable CVD risk factors in this setting. We find a substantial burden of modifiable risk factors, especially hypertension, diabetes, obesity (among women), and smoking (among men). These findings can help catalyze policy and clinical investments to improve the prevention, management, and control of CVD in these historically marginalized communities.

## Data accessibility statement

Statistical code is available at the Harvard Dataverse (https://doi.org/10.7910/DVN/KHWHPU). Survey data will be made available pursuant to the NIH data sharing policies.

## Additional File

The additional file for this article can be found as follows:

10.5334/gh.1171.s1 Appendix. Supplemental Methods and Results.
