# Supplementary material for: Prevalence of Hypertension, Diabetes, and Other Cardiovascular Disease Risk Factors in Two Indigenous Municipalities in Rural Guatemala: A Population-Representative Survey
Source: Glob Heart. 2022 Nov 22;17(1):82. doi: 10.5334/gh.1171 (PMC9695220; doi:10.5334/gh.1171)
Supplement: Appendix. — Supplemental Methods and Results. [file gh-17-1-1171-s1.pdf]

APPENDIX

	Total (n=806)	Male (n=280)	Female (n=526)
**Characteristic**		**Prevalence, % (95% CI)**	
Diabetes			
HbA1c ≥6.5%	9.5 (7.2 to 12.6)	9.2 (5.8 to 14.3)	9.9 (7.3 to 13.4)
HbA1c ≥6.5% or self-report of diagnosis	12.5 (9.6 to 16.1)	12.5 (8.3 to 18.4)	12.5 (9.3 to 16.6)
Hypertension			
≥140/90 mmHg	10.6 (8.0 to 14.0)	11.0 (7.3 to 16.1)	10.2 (7.2 to 14.4)
≥140/90 mmHg or self-report of diagnosis or using anti-hypertension medication	20.3 (17.1 to 23.9)	17.3 (12.6 to 23.3)	23.2 (18.4 to 28.7)
≥130/80	32.3 (26.9)	37.7 (29.6 to 46.6)	27.1 (22.0 to 33.0)
≥130/80 mmHg or self-report of diagnosis or using anti-hypertension medication	37.4 (32.6 to 42.4)	40.8 (33.2 to 48.7)	34.1 (28.3 to 40.4)
BMI			
Underweight (BMI <18.5)	3.4 (1.7 to 6.7)	5.3 (2.2 to 12.1)	1.6 (0.9 to 3.0)
Healthy weight (BMI 18.5 to 24.9)	33.6 (28.1 to 39.6)	36.2 (28.2 to 45.0)	31.1 (25.3 to 37.5)
Overweight (BMI 25.0 to 29.9)	39.3 (33.4 to 45.5)	41.7 (33.8 to 50.0)	37.0 (29.6 to 45.0)
Obese (BMI ≥30)	23.7 (19.4 to 28.6)	16.9 (10.6 to 25.7)	30.3 (25.1 to 36.1)
Smoking			
Current	10.7 (7.8 to 14.5)	20.5 (14.7 to 27.8)	1.2 (0.4 to 3.0)
Lifetime	32.2 (27.8 to 37.0)	57.8 (49.0 to 66.1)	7.5 (5.2 to 10.7)
High alcohol use			
> 2 days of drinking alcohol per week	0.9 (0.5 to 1.6)	1.3 (0.7 to 2.7)	0.4 (0.2 to 1.3)

Supplementary Table S1: Crude prevalence of modifiable cardiovascular disease risk factors using base and alternative definitions

Prevalence estimates were survey weighted with 95% confidence intervals in parentheses. The term crude refers to prevalence values in this table that are not age standardized to the WHO standard population. BMI=body mass index. CI=confidence interval. HbA1c=hemoglobin A1c. mmHg = millimeters of mercury.

Age (years)	Prevalence of 1 or more CVD risk factor, % (95% CI)	Prevalence of 2 or more CVD risk factor, % (95% CI)	Prevalence of 3 or more CVD risk factor, % (95% CI)	Prevalence of 4 or more CVD risk factor, % (95% CI)
18-29	36.3 (28.6 to 44.8)	4.5 (2.2 to 9.2)	0	0
30-39	45.3 (35.1 to 55.9)	10.2 (5.6 to 18.1)	0.4 (0.1 to 1.7)	0
40-49	65.6 (57.0 to 73.4)	17.3 (10.3 to 27.6)	3.7 (1.6 to 8.3)	0
50-59	69.0 (55.7 to 79.8)	36.2 (26.3 to 47.5)	7.9 (3.6 to 16.7)	0.4 (0.1 to 3.1)
60-69	61.2 (38.8 to 79.7)	31.8 (18.0 to 49.8)	8.2 (3.2 to 19.6)	0
≥70	46.3 (26.6 to 67.2)	23.2 (10.6 to 43.6)	0.6 (0.1 to 4.4)	0

Supplementary Table S2: Prevalence of co-occurring modifiable cardiovascular disease risk factors by age

The four modifiable CVD risk factors include obesity, defined as body mass index of ≥30 kg/m^2; hypertension, defined as ≥140/90 mmHg or self-report of diagnosis or using anti-hypertension medication; diabetes, defined as HbA1c ≥6.5% or self-report of diagnosis; and smoking, defined as a response of ‘current’ on a smoking questionnaire with options of never, former, or current. Prevalence estimates are survey weighted. These values also are depicted in Figure 2. CI=confidence interval. CVD = cardiovascular disease.

Supplementary methods: BP Measurement protocol

CONCEPT

Arterial Pressure (P/A): the resistance made by the arterial vessels of the human body against the force exerted by the passage of blood when expelled from the heart.

Systolic Blood Pressure: When the heart pushes blood into the artery.

Diastolic Blood Pressure: Period of relaxation of the heart.

For an adequate measurement of blood pressure, the following must be taken into account:

THE EQUIPMENT

The machines used to measure blood pressure are called sphygmomanometers and there are three types: mercury, aneroid and digital. In this study we will use digital sphygmomanometers.

THE BRACELET

It is a strip of cloth with a bag at one end and Velcro or metal hooks at the other for fastening. This bag has a rubber bladder or cuff attached. (Note: Any damage to the Velcro or fixing hooks must be reported immediately).

THE RUBBER BLADDER OR CUFF

The cuff, when inflated, has the function of compressing the blood vessels. The standard cuff is 11 X 23 cm, but there are three different sizes, which fit different arm circumferences or different age groups (infant, pediatric, adolescent, adult, obese). From the middle part of the sleeve comes a tube connected to the base of the device that includes the digital pressure gauge, which must be checked periodically to establish that there are no air leaks.

Ideally the cuff should cover 80% of the mid-circumference of the arm. Inaccurate readings may be obtained when the standard bladder or cuff is used and the mean arm circumference is greater than 29 cm. A cuff that is too small can overestimate blood pressure (false positives) and a cuff that is too large can underestimate blood pressure (false negatives).

Average arm circumference (cm)	Average cuff size	
	Width (cm)	Length (cm)
17–29	11	23
30–42	12.5	35

THE PATIENT

Blood pressure may be falsely elevated in the following circumstances:

- FEAR: The patient may be afraid of being sick, or fear that the result of the test will be unfavorable, that is, that his blood pressure will be high or low. Fear that the person taking the blood pressure may scold him, feel anguish if he perceives that the person

taking the pressure does not have the skills to do so or does so abruptly or without courtesy.

To reduce fear in the patient, treat him in a familiar, friendly, calm way, not in an accelerated way. Try to calm the patient, inform him that this is a routine examination that is done to all people, explain everything you are going to do to take the blood pressure (for example: correct position, rest, placement of the cuff, repetition of measurements).

- COLD: Cold can be the cause of high blood pressure values. Try to have your blood pressure taken at a comfortable room temperature.
- FULL URINARY BLADDER: Higher blood pressure values can be found when the patient is holding the urge to urinate. Before taking the blood pressure ask the patient if he does not want to urinate, if the answer is yes, ask him to empty his bladder.
- EXERCISE: It is likely that you will find higher blood pressure values if the measurement is made after the patient has performed some type of exercise or physical effort. Ask the patient to rest for 5 to 10 minutes, offer him a chair, and tell him to relax and rest.
- OBESITY: (Mean arm circumference greater than 29 cm.) Very small cuffs can give high readings for people with a lot of adipose tissue. Select the appropriate cuff for each patient, remember that the cuff or bladder should cover 80% of the circumference of the arm.

CONDITIONS FOR MEASUREMENT OF BLOOD PRESSURE

- Ask the subject to sit in a comfortable place and explain that to take the P/A they must remain seated for the first 10 to 15 minutes of the interview.
- Explain to the subject everything that he is going to do to take the P/A: clothing, correct position, placement of the cuff and the noise of the digital sphygmomanometer.

- Try to make the room quiet, try not to have people talking, children crying or screaming or the radio on. The quiet of the room helps the person relax.
- The person must be in a sleeveless shirt or blouse, otherwise ask them to remove their shirt, t-shirt, blouse, or sweater. Falsely low P/A can be found when clothing is too tight around the arm. If you decide to roll up your shirt or blouse, do it with delicacy and respect. Make sure that the cuff does not press on the soft tissues of the arm and that it does not interfere with placing the cuff. If so, ask the patient to remove their blouse or shirt.
- The P/A shots will be taken with the patient seated comfortably in a chair with a backrest and with a straight back.
- The measurement will be made on the left arm, which should be relaxed, somewhat separated from the body and with the middle of the arm at heart level. The position of the arm should allow the cuff to be placed without difficulty.
- The forearm should be semi-flexed, with the palm of the hand facing up, lying on a table or support (a pillow, a book or another) that helps maintain the relaxed position of the arm and the proper height. The body should not put force or weight on the arm or compress the cuff.
- Avoid letting your arm hang to the side of your body, this raises P/A by up to 10 mmHg.

MATERIAL AND EQUIPMENT

- Blood pressure cuff
- Chair with back
- Form for annotations

PROCEDURE

1. Palpate and record the radial pulse. It is located on the inner aspect of the forearm and on the wrist joint and is perceived with the index and middle fingers. Palpate the brachial pulse. This is easily palpated by making light pressure with the index and middle fingers on the inside of the elbow crease. Palpation of the brachial pulse is important to identify the exact place where the sphygmomanometer sensor should be placed.
2. Fit the cuff around the bottom of the cuff, it should be 2 cm above the elbow crease or 5 cm. above the vertex of the elbow and maintaining the same horizontal plane all around the arm. The cuff should be snug, not tight, but not loose either. If the cuff is loose, the pressure readings will be falsely high. Check that there is no clothing left under the cuff.
3. The cuff should cover the entire circumference of the arm, with its midpoint over the brachial artery. To ensure proper cuff placement, the bladder tube should be above and in the middle of the elbow crease, not kinked or pinched.
4. The blue button (start) is pressed. You will immediately hear a noise, this indicates that the cuff is inflating. Eventually it may happen that the device stops inflating and a few seconds later resumes inflation, indicating that the pressure level knob should be set to a higher level on this subject.
5. After inflating the cuff you will hear Beep, Beep and three numbers will appear on the digital display in descending order, the first corresponds to the systolic pressure, the second to the diastolic pressure and the third to the pulse.
6. Record the systolic, diastolic, and pulse pressure values on the form. Be careful because 1 minute later the digital display will be off. If you do not record the data on the form because the screen turned off, repeat the procedure five minutes later.
7. The procedure will be repeated two other times, with an interval of five minutes between each one, writing down the values obtained; If the difference between the values of the second and third measurements is greater than 10 mmHg, a fourth measurement will be taken five minutes later.

8. Remove the cuff and give the person the results of the blood pressure measurement.
